# Development of an Antibacterial Poly(Lactic Acid)/Poly(ε-Caprolactone)/Tributyl Citrate Film Loaded with *Staphylococcus aureus* Bacteriophages Using a Sodium Alginate Coating

**DOI:** 10.3390/ijms26167793

**Published:** 2025-08-12

**Authors:** Seulgi Imm, Jaewoo Bai, Yoonjee Chang

**Affiliations:** 1 Department of Food and Nutrition, College of Science and Technology, Kookmin University, Seoul 02707, Republic of Korea; 2 Food Science and Technology, Seoul Women’s University, Seoul 01797, Republic of Korea

**Keywords:** antibacterial packaging, bacteriophage, poly(lactic acid), poly(ε-caprolactone), biodegradable

## Abstract

Biodegradable poly(lactic acid) (PLA)/poly(ε-caprolactone) (PCL) composite films were prepared with a compatibilizer (tributyl citrate, TBC) using a solvent casting method. Incorporation of 5% TBC (*w*/*v*, of PCL weight) improved tensile strength and elongation at break (21.93 ± 2.33 MPa and 21.02 ± 1.54%, respectively) and reduced water vapor permeability (from 0.12 ± 0.01 to 0.098 ± 0.01 g·mm·m^2^·h·kPa), indicating improved compatibility between PLA and PCL. *Staphylococcus aureus* phage PBSA08 demonstrated rapid and persistent bacteriolytic activity for up to 24 h, suggesting its potential as a promising antibacterial biological agent. To impart antibacterial properties to the developed PLA/PCL/TBC film, PBSA08 was loaded into sodium alginate (SA) and coated on the film surface. The optimal composition was 3% (*w*/*v*) SA and 3% (*w*/*v*) glycerol, which exhibited suitable dynamic behavior as a coating solution and excellent adhesion to the film surface. The phage-coated antibacterial films demonstrated progressive and significant inhibition against *S. aureus* starting from 10 to 24 h, with controlled phage-release properties. Overall, the developed active film might exert sustained and remarkable antibacterial effects through controlled release of biological agents (phage) under realistic packaging conditions.

## 1. Introduction

The creation of natural and sustainable polymer-based resources as based on polymers as workable substitutes for plastics derived from fossil fuels has garnered interest for a variety of reasons [1,2]. One of the most promising bio-based polyesters for biodegradability in food packaging is poly(lactic acid) (PLA), which is currently the subject of intensive research [3]. PLA has outstanding processability, high barrier qualities, and mechanical capabilities for a variety of applications in addition to being thermoplastic and biodegradable [4]. Nevertheless, there exist several limitations, including inherent brittleness, low flexibility, and slow crystallization [5]. Consequently, the toughening modification of PLA is essential to develop an effective packaging system. Combining PLA with flexible polymers like poly(butylene succinate) (PBS), poly(butylene adipate-co-terephthalate) (PBAT), and poly(ε-caprolactone) (PCL) is the most efficient way to build PLA-based packaging while enhancing PLA’s characteristics [6].

In particular, the combination of PLA with PCL has been proposed to develop novel biomaterials. In contrast to PLA’s high modulus TS and low EAB, PCL exhibits low tensile strength (TS) and high elongation at break (EAB) [7]. However, due to their high interfacial tension, which produces coarse morphologies and subpar mechanical characteristics, PLA and PCL are regarded as incompatible polymers. Hence, better dispersion and compatibility of this immiscible polymer are of prime importance for preparing PLA/PCL blends.

Many investigations on different compatibilizers to improve PLA and PCL miscibility have been carried out. Specifically, tiny molecular chemicals like dicumyl peroxide (DCP), glycidyl methacrylate (GMA), and triphenyl phosphite (TPP) are added to the PLA/PCL blend to improve its toughness and minimize the size of dispersed phases [8,9,10]. Nonetheless, these agents are difficult to manufacture and highly toxic, rendering them unsuitable for incorporation into packaging materials. By causing the PLA and PCL mix to cross-link, the eco-friendly low-molecular-weight ester tricytyl citrate (TBC) can increase the blend film’s ductility and impact strength [11]. Moreover, TBC interacts polarly with PLA’s ester group to show great solubility [12]. Consequently, adding TBC may enhance PLA/PCL composite film miscibility, which would enhance the film’s mechanical and barrier qualities.

Bacteriophages (phages) are emerging as natural biocontrol agents of food microbial contamination [13]. The most common organisms on Earth are called phages, which are viruses that exclusively infect bacteria [14]. Phages are regarded as prospective biological agents in the food business because to their great infectivity against antibiotic-resistant bacteria and extraordinary host specificity [15].

In fact, various active packaging containing phages have been recently investigated, including hydrogels, films, coatings, and capsules [16,17,18]. Nevertheless, from an industrial perspective, commercial films are primarily manufactured using polymer technology processes such as extrusion [19], in which most antibacterial substances tend to lose activity under conditions of high temperature, rapid shear rate, and high pressure, possibly reducing their efficacy against microorganisms [20]. Therefore, new alternative methods have been applied to introduce activators such as phages into the packaging industry. For example, several studies have successfully incorporated bacteriophages into industrially scalable active packaging via techniques such as flexography, extrusion, and spraying [21,22,23]. These strategies demonstrate the feasibility of applying phages to commercial-scale food packaging systems. In this study, we focus on integrating sodium alginate-entrapped phages into a PLA/PCL/TBC matrix via bar-coating, a method simulating roll-to-roll coating to control using *Staphylococcus aureus*. For this purpose, we added the compatibilizer TBC to the PLA/PCL composite film and evaluated the mechanical, moisture, and surface properties. Moreover, a sodium alginate coating with glycerol that was used as a carrier for the phage was applied to the developed film surface using a bar-coating method. We then analyzed the rheological properties and surface adhesion of sodium alginate to optimize the coating composition. Finally, the phage was loaded onto the sodium alginate coating and immobilized on the film surface, and its antibacterial effects against *S. aureus* was monitored for 24 h.

## 2. Results and Discussion

### 2.1. Determination of the PLA/PCL Film Composition

Mechanical properties such as TS and EAB are considered important factors in packaging with respect to breaking prohibition and puncture resistance throughout the packaging process [24]. Table S1 shows the mechanical properties of neat PLA, PCL, and PLA/PCL composite films. Neat PLA formed a robust film with a relatively high TS of 30.89 ± 0.24 MPa; however, neat PCL formed a mechanically weak film with a TS of 10.84 ± 0.79 MPa. Moreover, the TS of PLA/PCL blend films decreased considerably with increasing PCL concentration (*p* < 0.05). PCL improves ductile properties (similar to that of plasticizers), resulting in lower TS. Furthermore, the EAB of the PLA/PCL composite film increased up to 33.08%, which significantly enhanced the toughening effect. Nonetheless, when PCL was excessively added (PLA_60_PCL_40_), there was a decrease in EAB. It is known that when the polymer is immiscible, the apparent phase separation of the continuous phase (PLA) increases with increasing minority of polymer domains (PCL), causing a significant decrease in elongation [25].

**Figure 1:**
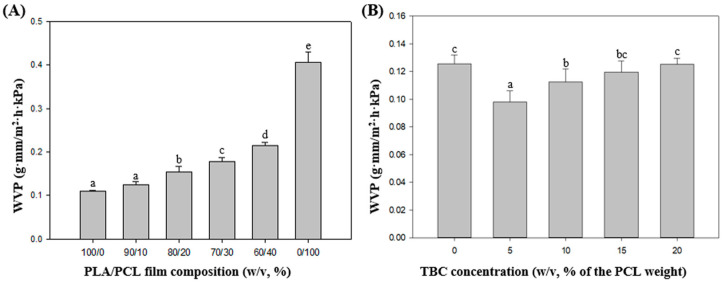
Water vapor permeability (WVP) of PLA/PCL films according to (**A**) PLA and PCL blend composition (*w*/*v*, %) and (**B**) compatibilizer TBC concentration (*w*/*v*, % of PCL weight). Each column shows the mean of three independent experiments, and error bars represent the standard deviation. Different letters indicate statistically significant differences (*p* < 0.05).

The significance of WVP for packaging depends on its role in preventing deteriorative reactions and microbial growth caused by water present inside the packaging [26]. The WVP values of neat PLA and neat PCL were 0.11 ± 0.01 and 0.39 ± 0.04 g·mm/m^2^·h·kPa, respectively (Figure 1A). The WVP of PLA gradually increased as the PCL blending ratio increased (*p* < 0.05). It is predicted that the system will be poorly compatible when the PCL content increases, causing more voids at the interface junction, intensifying the occurrence of water vapor permeation [27]. Meanwhile, the WVP of PLA_90_/PCL_10_ (0.11 ± 0.01 g·mm/m^2^·h·kPa) was comparable to that of the neat PLA film (0.12 ± 0.01 g·mm/m^2^·h·kPa). Thus, the blending ratio was finally determined with PLA_90_/PCL_10_, which improves the mechanical properties and maintains the moisture properties.

### 2.2. Determination of TBC Composition in the PLA/PCL Film

Table S2 and Figure 1B illustrate how the compatibilizer TBC affects the mechanical characteristics and WVP of PLA_90_PCL_10_ composite films. The TS of the film showed significant improvement with the increase in TBC content (*p* < 0.05), whereas the EAB of the film showed limited improvement. The significant contribution of TBC to the toughness of PLA/PCL might be due to improved interfacial compatibility between PLA and PCL, promoting chain extension and plastic deformation [28]. A previous study also reported similar results, wherein nano-lignin was grafted on the PLA chain as an interfacial compatibilizer, resulting in increased PLA flexibility and toughness [29].

Regarding WVP, the inclusion of TBC effectively improved the film’s moisture barrier behavior. Regarding WVP, the inclusion of TBC significantly improved the film’s moisture barrier performance. When 5% TBC (*w*/*v*, of PCL weight) was added, the WVP decreased from 0.12 ± 0.01 to 0.098 ± 0.01 g·mm/m^2^·h·kPa. However, with increasing concentrations of TBC, the barrier performance of PLA/PCL composites gradually decreased (*p* < 0.05). In general, chain extension and cross-linking increase the entanglement of molecular chains and improves barrier properties but exhibit the opposite tendency [30]. This result suggests that an appropriate amount of TBC can effectively improve matrix compatibility, whereas excessive addition can reduce the compatibility of PLA and PCL, and generate voids in the interface bond, which is a major cause of barrier deterioration.

The goal of this study was to investigate the changes in film properties due to the addition of TBC and to determine the TBC concentration contributing to the optimal properties of the film. Therefore, the PLA_90_/PCL_10_ film with addition of 5% TBC (*w*/*v*, of PCL weight) (PLA_90_/PCL_10_/TBC_5_) with improved moisture barrier properties was selected as the optimal film composition. Hence, this study could serve as a reference for designing to develop films with improved miscibility by adding compatibilizers to immiscible polymers.

### 2.3. Film Characterization

#### 2.3.1. Optical Properties of Films

As a packaging material, highly transparent films can represent products clearly and completely, and opaque films can protect products from damage from light energy. Therefore, controlling transparency is a crucial aspect of packaging films. Table S3 shows the surface color of PLA, PCL, and PLA/PCL composite films (with and without TBC). High L-values (lightness) of 92.17 and 92.97 were shown by both PLA and PCL films, respectively. The brightness of the PCL film was higher than that of the PLA film, and the addition of PCL slightly increased the brightness of the PLA/PCL film (*p* < 0.05). Other than that, no obvious change was observed in color values depending on the single film, composite film, and the presence of TBC. This result was consistent with the appearance of the actual films (Table S3).

The films’ transparency and UV light-blocking capabilities are indicated by their optical transmittance at 660 nm (T660) and 280 nm (T280), respectively. The PLA film exhibited high transparency with a T660 of 90.7%, whereas the PCL film exhibited a relatively lower transparency with a T660 of 7.6%. The transparency of the PLA film decreased when blended with PCL. Moreover, the PLA film transmitted 90.7% of UV light (T280), whereas the PCL film demonstrated excellent UV light-blocking properties with a T280 of 0.94%. Even a small amount of PCL in PLA (PLA_90_/PCL_10_) significantly decreased the UV light transmittance to 44.59% (*p* < 0.05), emphasizing the potent UV protection capacity of PCL. The stability of the PCL structure and the absorption of UV light are caused by the presence of carbonyl groups; these groups are found in ester bonds and function as an energy sink by dissipating UV energy through electron delocalization [31]. Therefore, the optical transmittance results of PLA/PCL composite films suggested that incorporating a minor amount of PCL slightly compromised the transparency and improved the UV light absorption capabilities.

#### 2.3.2. Morphologies of Films

**Figure 2:**
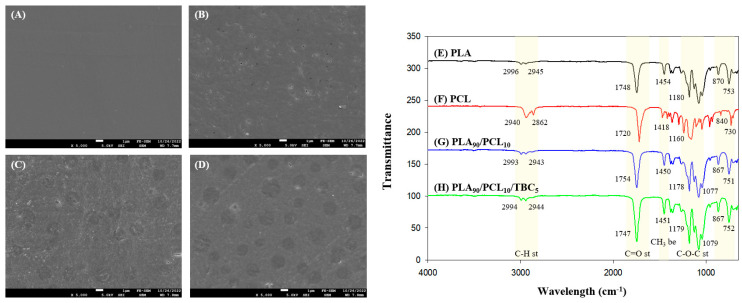
Scanning electron microscope (SEM) images and FTIR spectra of PLA, PCL, and PLA/PCL composite films (with and without TBC). The SEM images at 5000× magnification show the surface morphology of (**A**) PLA, (**B**) PCL, (**C**) PLA90/PCL10, and (**D**) PLA90/PCL10/TBC5. FTIR spectra of (**E**) PLA, (**F**) PCL, (**G**) PLA90/PCL10, and (**H**) PLA90/PCL10/TBC5, highlighting the characteristic peaks due to blending and compatibilizer addition.

The SEM micrographs of PLA, PCL, and PLA/PCL composite films (with and without TBC) are depicted in Figure 2A–D. The neat PLA film featured a smooth, even surface free of ridges, with a continuous phase (Figure 2A). The neat PCL film had a few micro-voids on the rough surface of the film (Figure 2B). When PLA was blended with PCL, partial phase separation was observed in the films, indicating low miscibility between the two components (Figure 2C,D).

In particular, the PLA/PCL composite film without TBC had a rough surface, and the PCL particles that were not sufficiently dispersed within the PLA were observed on the film surface. However, the film with TBC presented a relatively smooth surface and improved particle distribution throughout the polymer matrix by plasticizer. The low-molecular-weight compatibilizer TBC can easily penetrate the interface of the composite components and make them compatible to improve the smoothness of the overall film surface [32]. These results are consistent with previous research, wherein triethyl citrate and GMA improved the dispersion of PBAT in the PLA matrix [33,34]. Furthermore, the inclusion of TBC contributed to the removal of pores on the surface, which could change the film’s WVP. These results demonstrated that TBC changed the structure of the composite film, reducing the number of pores and lowering the WVP (from 0.1255 to 0.0980 g·mm/m^2^·h·kPa). Therefore, the presence of TBC caused an improvement in the miscibility and barrier properties of the PLA/PCL blend.

#### 2.3.3. FTIR Analysis

Figure 2E–H shows the FTIR spectra of PLA, PCL, and PLA/PCL composite films with and without TBC. Peaks of pure PLA observed at 2998, 2947, and 1751 cm^−1^ and those of pure PCL observed at 2942, 2867, and 1723 cm^−1^ correspond to C-H stretching vibration and C=O stretching vibration, respectively [35].

Moreover, peaks of pure PLA observed at 2998 and 1751 cm^−1^ and those of pure PCL observed at 2942 and 1723 cm^−1^ indicate CH_3_ bending vibration and C=O stretching vibration, respectively [36,37]. Peaks observed at 755 and 874 cm^−1^, respectively, correspond to the crystalline and amorphous phases of PLA, suggesting that PLA is composed of semi-crystalline forms [12].

Although the characteristic peaks of PLA and PCL were observed in the spectra of PLA/PCL composite films, there were some changes within the wavelength region or peak intensity. Compared with pure PLA, there was a peak position shift in the C=O stretching vibration region (1748–1745 cm^−1^), implying the involvement of the C=O group of PLA interacts with the terminal −OH groups of low-MW or partially degraded PCL via hydrogen bonding [37]. Furthermore, the PLA/PCL film with the addition of TBC demonstrated increased intensity at the C=O peak. The carbonyl groups (C=O) of citrate esters such as TBC could interact via hydrogen bond formation with terminal hydroxyl groups (−OH) in the primary structures of both PLA and PCL [12]. Nevertheless, the FTIR spectra of the PLA/PCL/TBC film showed no additional peak through those chemical interactions, presumably because of the small amount of TBC present in the sample. Additionally, the carbonyl groups of TBC may increase the peak intensity due to polar interactions with PLA/PCL, although no new peaks were observed due to the small TBC concentration.

### 2.4. Determination of the SA Coating Solution Composition

**Figure 3:**
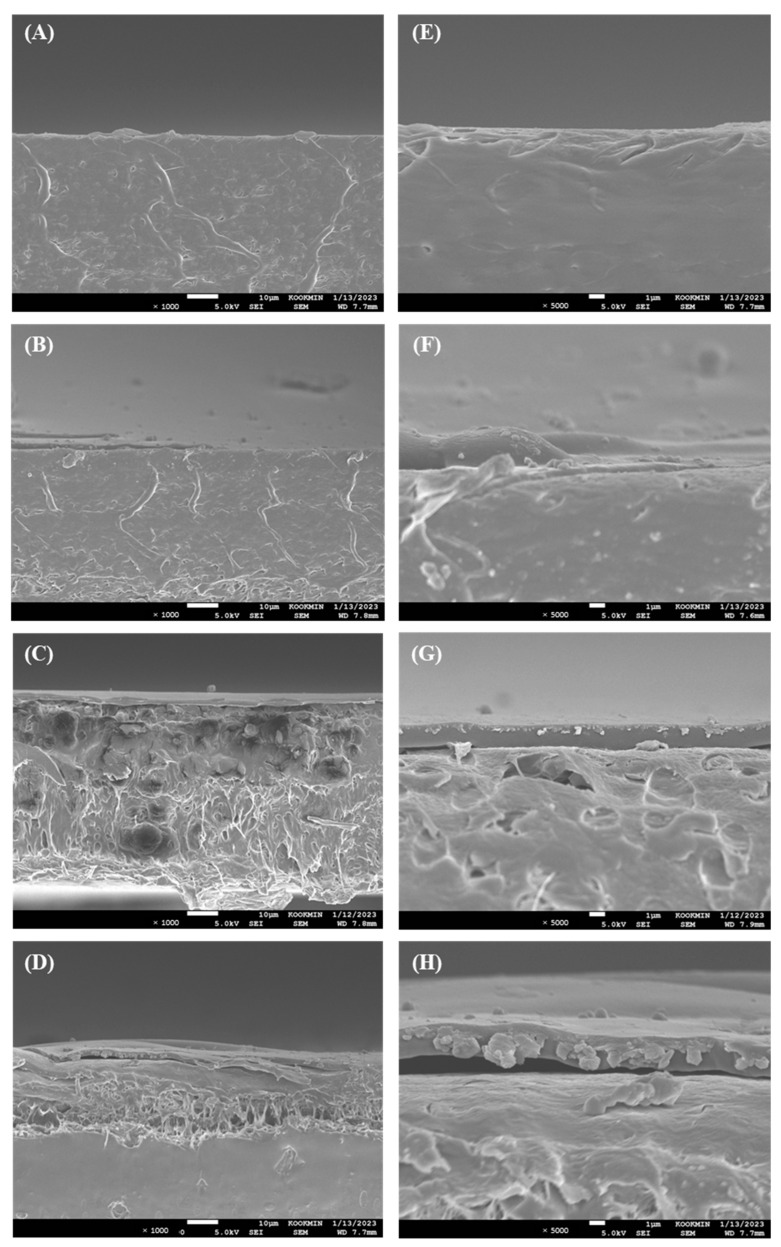
Scanning electron microscope (SEM) images of sodium alginate (SA)-coated PLA/PCL/TBC films at different SA concentrations. (**A**,**E**) Noncoated film; (**B**,**F**) 2% SA-coated film; (**C**,**G**) 3% SA-coated film; (**D**,**H**) 4% SA-coated film under different magnifications (1000× and 5000×, respectively).

Rheology of a solution is a crucial property in coating processing and application. To determine the rheological properties of the coating solution, the viscosity of SA solutions at different concentrations [2%, 3%, and 4% (*w*/*w*)] was evaluated. As demonstrated in Figure S1, all SA coating solutions displayed typical shear thinning flow characteristics of non-Newtonian fluids, with viscosity decreasing as shear rate increased due to network destruction [38]. Several coating solutions and paints exhibit these dynamic properties because solutions with this flow could maintain stable coatings under static conditions and uniformly coat with a constant viscosity at high shear rates. This rheological characteristic was consistent with previous reports on the viscosity of SA solutions at various concentrations [39,40]. We evaluated the shear rate from 1 × 10^2^ to 3 × 10^3^ (1/s), which corresponds to the range of shear rates applied to roller-coating and brushing for common industrial operations [41]. In this shear rate range, the viscosity increased significantly as the SA concentration increased. The 3% SA solution exhibited a uniform coating layer through drawdown coating. However, the 2% SA solution did not completely adhere to the film surface and exhibited an uneven coating layer due to a lack of viscosity. Moreover, the 4% SA solution was agglomerated as the applicator passed and bead-up on the film due to its high viscosity. This tendency was further examined by observing the cross section of the coated film by SEM (Figure 3). The 2% SA solution did not sufficiently adhere to the film surface, causing the solution to clump together and break the coating (Figure 3B,F). The 4% SA solution had a thickness of approximately 1.97 μm due to its high viscosity and was bead-up from the surface. Conversely, 3% SA solution demonstrated the maximum adhesion and a constant thickness of approximately 1.54 μm.

**Figure 4:**
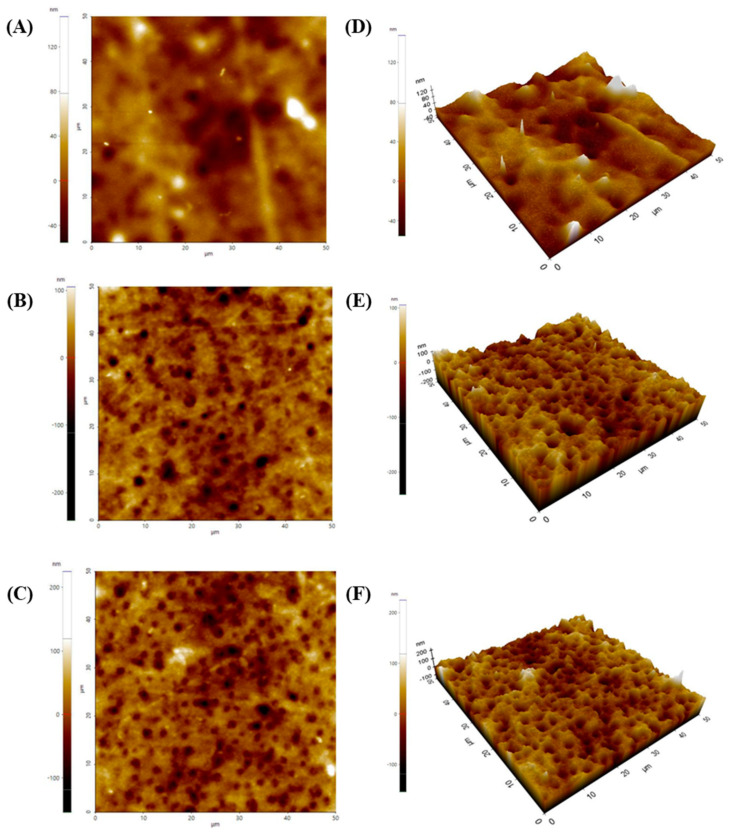
Atomic force microscopy (AFM) images of sodium alginate (SA)-coated PLA/PCL/TBC films at different SA concentrations. (**A**,**D**) 2% SA-coated film; (**B**,**E**) 3% SA-coated film; (**C**,**F**) 4% SA-coated film with different dimensions (2D and 3D, respectively).

The surface roughness of the coating layer was analyzed using Park Systems’ SmartScan software (Figure 4) and expressed as Rq value. The AFM images revealed that the 2% SA coating layer presented the smoothest and most uniform surface highlighted by the lowest surface roughness value (Rq: 7.497 nm). The heterogeneous pattern became more distinct with an increase in SA concentration (Rq: 16.922–22.335 nm). An obvious increase in roughness may occur due to the solid content and aggregate structure formation with increasing SA concentrations. However, the 3% SA solution was finally selected as an optimal coating formulation because the most suitable film adhesion and uniform coating layer were observed in the 3% SA-coated layer.

### 2.5. Phage Characterization

#### 2.5.1. Morphological Analysis

**Figure 5:**
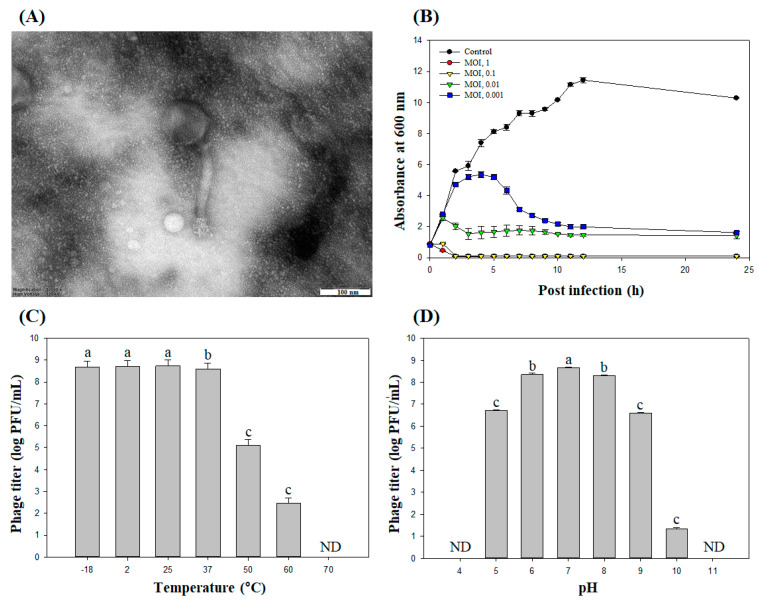
(**A**) Transmission electron micrograph (TEM), (**B**) bacteriolytic effect, (**C**) thermal stability, and (**D**) pH stability of phage PBSA08. Each column depicts the mean of three independent experiments, and error bars represent the standard deviation. Different letters indicate statistically significant differences (*p* < 0.05). ND, not detected.

The TEM micrograph revealed that phage PBSA08 had a conformation with an icosahedral head and rigid contractile tails (Figure 5A). The phage’s head diameter was 95 ± 3 nm (*n* = 5). The tail measured 168 ± 6 nm (*n* = 5) in length and 21 ± 1 nm (*n* = 5), in width, respectively. These structural characteristics indicate that the phage belongs to the *Myoviridae* family in the *Caudovirales* order [42].

#### 2.5.2. Host Range and Efficiency of Plating Analysis

The spotting assay revealed that phage PBSA08 was capable of infecting 100% (14/14) of the tested *S. aureus* strains (Table 1), but it did not infect any other strains, suggesting that phage PBSA08 has a high level of host specificity. The relative EOP analysis conducted on 18 strains that were susceptible to phages indicated that phage PBSA08 had high productive infectivity against nine *S. aureus* strains (1 ≤ EOP), medium infectivity against four strains (0.5 ≤ EOP < 1), and low infectivity against four strains (EOP < 0.5).

#### 2.5.3. Bacteriolytic Efficacy Against *S. aureus*

The bacterial growth inhibition activity of phage PBSA08 against *S. aureus* at various MOIs (1, 0.1, 0.01, and 0.001) (Figure 5B) was evaluated and found that the phage-treated groups exhibited remarkably suppressed bacterial growth at all MOIs. Treatment with the phage at MOIs of 1 and 0.1 resulted in rapid antibacterial efficacy within 1 h. Even at a very low MOI (0.001), the phage-treated groups demonstrated persistent inhibitory activity that lasted up to 24 h as compared to the control. However, previous studies have reported that the *S. aureus* phages SA [43] and SA97 [44] exhibited antibacterial ability that was maintained for only approximately 10–11 h. These data indicated that PBSA08 possesses strong bacteriolytic efficacy, suggesting its high potential as an efficient biological agent for food application.

#### 2.5.4. Temperature and pH Stability Assessment

It is essential to confirm phage stability under a range of stress conditions for food applications. Therefore, we determined the stability of phage PBSA08 under different temperature and pH conditions. In the temperature stability test, the phage PBSA08 was moderately stable from −18 °C to 37 °C; however, its viability significantly diminished from 50 °C to 60 °C (3.0- and 6.1-log PFU/mL reduction, respectively), and it was entirely inactivated at 70 °C (Figure 5C). Regarding pH stability, the phage was moderately stable at neutral range (pH 6–8); however, its viability significantly diminished at pH 5 and pH 10 (1.9- and 6.9-log PFU/mL reduction, respectively), and it was entirely inactivated at pH 4 and pH 11 (Figure 5D). This thermal and pH stability of PBSA08 would be useful for application as a biocontrol agent.

#### 2.5.5. Phage Genome Analysis

**Figure 6:**
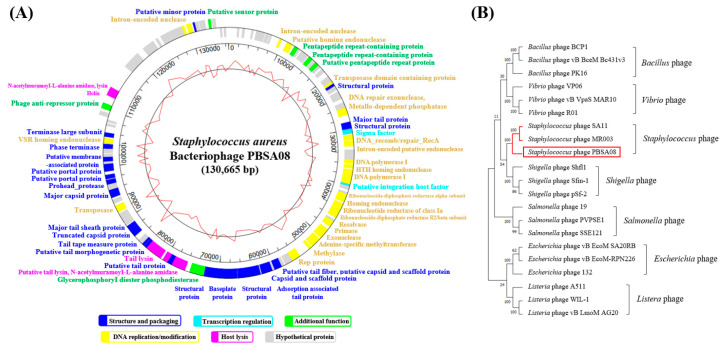
(**A**) Genome map of phage PBSA08. The outer circle indicates the predicted open reading frames (ORFs), and specific colors represent the functional category. The inner circle with a red line indicates the GC content. Scale units are base pairs. (**B**) Phylogenetic analysis of phage PBSA08 (red box). The phylogenetic tree based on the sequences of terminate large subunit represents the homology between phage PBSA08 and other phages. Numbers next to the branches indicate the bootstrap value.

The complete genome of phage PBSA08 revealed 136,673 bp with a GC content of 30.01% and encoding 119 ORFs (Figure 6A).

Of these 119 ORFs, 63 (53%) were identified as a special functional protein. These ORFs were classified into five representative functional clusters, including structure and packaging (structural protein, major tail protein, and capsid and scaffold protein), transcription regulation (sigma factor and putative integration host factor), DNA replication/modification (intron-encoded nuclease and DNA repair exonuclease), host lysis (holin, *N*-acetylmuramoyl-l-alanine amidase, and tail lysin), and an additional function (glycerophosphoryl diester phosphodiesterase and putative sensor protein). Moreover, no lysogenic genes (e.g., *cro*, *c*I, and integrase) were identified in phage PBSA08, suggesting that it is a lytic phage and can be employed safely as a biocontrol agent.

The results of the phylogenetic analysis based on the terminate large subunit (ORF97) of the phage revealed that *Staphylococcus* phages SA11 and MR003 (GenBank accession no. JX194239 and AP019522, respectively) were clustered with phage PBSA08 (Figure 6B). This finding indicated that phage PBSA08 is evolutionarily closely related to SA11 and MR003, both of which belong to the *Staphylococcus* phage.

### 2.6. Antibacterial Efficacy of SA-Coated PLA/PCL/TBC Films Containing S. aureus Phage PBSA08

**Figure 7:**
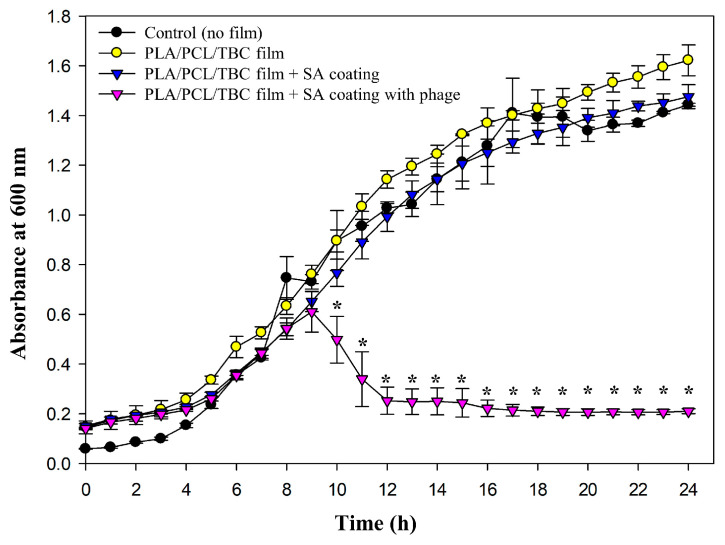
The growth of *S. aureus* at 37 °C for 24 h with different antibacterial film treatments. Black, control (no film); yellow, PLA/PCL/TBC film; blue, PLA/PCL/TBC film + SA coating; pink, PLA/PCL/TBC film + SA coating with phage. Error bars indicate the standard deviation. The asterisk (*) indicates the level of *p* < 0.05, revealing a significant difference between the control and phage-treated group.

The antibacterial activity of SA-coated PLA/PCL/TBC films containing PBSA08 was evaluated against *S. aureus* (Figure 7).

The bacteria showed remarkable growth in all experimental groups by 9 h with an upward curve. The phage-coated film group exhibited no significant bactericidal efficacy until 9 h (*p* < 0.05). These findings indicated delayed antibacterial properties compared with the challenge assay results of PBSA08 (Figure 5B), suggesting a controlled release of the phage from the SA matrix. It has been previously demonstrated that the T7-like *Podoviridae* phage ϕIBB-PF7A (head diameter of approximately 63 nm and tail size of approximately 13 × 8 nm) embedded inside the SA film was rapidly released from the SM buffer within 2–3 min [45]. Conversely, PBSA08 is a *Myoviridae* phage with a relatively large size (head diameter of approximately 95 nm and tail size of approximately 168 × 21 nm), possibly delaying its release from the SA matrix. However, the phage-dependent reduction in bacterial growth was significantly more pronounced with a stationary phase average absorbance of 0.210 after 24 h compared to 1.44, 1.62, and 1.47 for the control (no film), PLA/PCL/TBC film, and PLA/PCL/TBC film with SA coating, respectively. According to published standard curves for *S. aureus*, this absorbance is consistent with the inhibition of bacterial growth of approximately 5-log CFU/mL [46]. Overall, these results demonstrated a significant reduction in *S. aureus* growth, revealing that phages provide an efficient antimicrobial activity when incorporated into SA. Although only the film coated with 3% SA was evaluated for antibacterial efficacy, the surface and structural properties of films coated with 2% and 4% SA (Figure 3 and Figure 4) suggest that phage-release rates may be influenced by the physical structure of the phage thickness and roughness of the alginate matrix. Further investigation is needed to quantify release behavior at different SA concentrations.

To our knowledge, this is the first study in which phages were loaded into SA and coated on the PLA/PCL/TBC film surface. This approach might exert a sustained and substantial antibacterial effect against *S. aureus* through controlled release of biological agents under actual packaging conditions.

## 3. Materials and Methods

### 3.1. Materials

PLA pellet (grade 2003D) was supplied by Natureworks (Blair, NE, USA), and PCL pellet was supplied by Xiaogan Esun New Material Co., Ltd. (Xiaogan, China). The sources of chloroform and TBC were Sigma-Aldrich Co., Ltd. (St. Louis, MO, USA). Sodium alginate (SA) and 99% glycerol were acquired from Daejung Chemicals Co., Ltd. (Gyeonggi, Republic of Korea).

### 3.2. Preparation of PLA/PCL/TBC Composite Films

Solvent casting was used to prepare PLA/PCL composite films of various compositions. The PLA/PCL blending ratios were 100/0%, 90/10%, 80/20%, 70/30%, 60/40%, and 0/100% (*w*/*v*), each sample was dissolved using a 1:20% (*w*/*v*) solvent ratio in chloroform. Subsequently, TBC was added to the PLA/PCL solution at different weight percentages [0.5%, 1%, 3%, and 5% (*w*/*v*)] of PCL weight. After 6 h of stirring at room temperature (RT), the mixture was transferred to a glass Petri dish (150 × 150 mm) that had a Teflon layer attached to it. All films were dried for an additional 6 h at 50° C in a drying oven to eliminate any last traces of solvent, following 18 h of RT drying. Before the studies, the films were allowed to condition for at least 24 h at 25 °C and 50% relative humidity (RH).

### 3.3. Sodium Alginate Coating Preparation and Treatment

SA coating solutions plasticized with 3% (*w*/*v*) glycerol of various concentrations were prepared [2%, 3%, and 4% (*w*/*v*)]. Briefly, SA and glycerol were dispersed in distilled water, and the mixture was heated over 60 °C under agitation until the solids were completely dissolved. Following adequate cooling, the coating solutions were applied to the PLA/PCL/TBC film by using an applicator (bar coater, RD Specialties, Inc., New York, NY, USA) to dispense them onto the film surface at a wet thickness of 13.72 μm. The coated films were then dried for 4 h at 25 °C. For preparing the antibacterial film, the phage was additionally loaded in the SA/glycerol solution at 1/10% (*v*/*v*) and then coated on the film (10^6^ PFU/cm^2^).

### 3.4. Characterization of the Films

#### 3.4.1. Film Thickness and Mechanical Properties

The thickness of the films was measured at five random sites using the clearance gauge 547-401 (Mitutoyo, Kawasaki, Japan). The mechanical, moisture barrier, and optical characteristics were ascertained by taking the mean of each value. A texture analyzer (UTA-500N, Yeonjin, Seoul, South Korea) was used to quantify TS and EAB. Film samples were divided into 15 × 100 mm pieces and positioned in between the machine’s grasp heads. The crosshead speed and the gauge length were 50 mm/min and 60 mm, respectively.

#### 3.4.2. Water Vapor Permeability

The ASTM E96 technique was used to measure the film’s WVP gravimetrically. After cutting the films into a 65 mm diameter circle, they were hermetically sealed onto a circular cup that held anhydrous calcium chloride. Every film was pre-weighed and kept at 25 °C and 50% RH. After measuring the sample’s final weight after 24 h, the WVP value was computed using as follows:

$$WVP\left(g/m^{2}\cdot h\cdot kPa\right)=(∆W\cdot x)/(A\cdot t\cdot ∆P)$$

where ∆W is the difference in the cup’s weight (g) after 24 h, x is the mean thickness of the films (mm), A is the exposed permeation area of the films (m^2^), t is the duration (h), and ∆P is the partial difference in water vapor pressure across the two sides of the film (Pa).

#### 3.4.3. Optical Properties

The films’ L* (lightness), a* (redness), and b* (yellowness) were measured with a colorimeter (CR-400 Chroma Meter, Konica Minolta Sensing, Inc., Osaka, Japan) to assess the surface color. The apparatus was configured with a 2° observer angle and a D65 illuminant. Three randomly selected places on the film surface were used to test the color values after the films were placed on the standard white plate (L* = 93.35, a* = −0.23, and b* = 4.34). The following formula was used to determine the total color difference (ΔE):

$$\Delta E=\sqrt{{\left({∆L}^{*}\right)}^{2}\;+\;{\left({∆a}^{*}\right)}^{2}+\;{\left({∆b}^{*}\right)}^{2}}$$

where ΔL*, Δa*, and Δb* represent the color differences between the standard white plate and the control film (PLA). A spectrophotometer (SP-UV 300, PerkinElmer, Inc., Boston, MA, USA) was used to measure the transmittance at 600 nm to observe transparency. Transparency was evaluated at three different random locations on each rectangular piece of film.

#### 3.4.4. Scanning Electron Microscopy Analysis

The microstructure of the films was investigated with a scanning electron microscope (JSM-7610F, JEOL Ltd., Tokyo, Japan). The samples were coated with Pt/Pd for 30 s after being positioned on carbon tape. Film samples were viewed at a magnification of 5000×, and the coated films were viewed again at a magnification of 1000× while being accelerated at a voltage of 5.0 kV.

#### 3.4.5. Fourier-Transform Infrared (FTIR) Spectroscopy

An attenuated total reflectance device was fitted to a Fourier-transform infrared (FTIR) spectrometer (Cary 630, Agilent Technologies Inc., Santa Clara, CA, USA) for the analysis of the films’ FTIR spectra. The analysis was carried out using 64 scans at a resolution rate of 4 cm^−1^ in the spectral band of 4000–650 cm^−1^.

### 3.5. Characterization of the SA Coating Solution

#### 3.5.1. Viscosity Analysis

For determining the rheological properties of the coating solution, viscosity was analyzed according to the standard testing technique according to ISO 3219-1:2021, Rheology—Part 1: Vocabulary and symbols for rotational and oscillatory rheometry [47]. The viscosity of SA coating solutions [2%, 3%, and 4% (*w*/*v*)] plasticized with 3% (*w*/*v*) glycerol was measured with a rotational rheometer (ARES-G2, TA Instruments, New Castle, DE, USA) with a 40-mm parallel-plate geometry and a gap of 0.5 mm. The analysis was conducted in a shear rate range of 1–3000 1/s at 25 °C, and the viscosity was calculated according to the following formula:

$$Viscosity\left(Pa\cdot s\right)=Shear\;stress\left(Pa\right)/Shear\;rate(1/s)$$

#### 3.5.2. Atomic Force Microscopy

The coated films with varying SA concentrations were observed by AFM. First, the SA-coated films were sliced into pieces (10 × 10 mm) using a small sharp knife and stuck on the stage. Each sample was scanned using an NX10 AFM (Park Systems, Suwon, South Korea) with the tapping mode. The root mean square of roughness (Rq) value was used to assess the surface roughness of each sample.

### 3.6. Bacteriophage (Phage) Preparation

#### 3.6.1. Bacterial Strains and Culture Conditions

*S. aureus* ATCC 29213 was used as the host bacterial strain in this study. The phage PBSA08 (BP-6008) was obtained from the bacteriophage bank at Hankuk University of Foreign Studies in Korea. *S. aureus* strains were cultured aerobically in tryptic soy broth (TSB) and tryptic soy agar (TSA) at 37 °C. The other strains were cultured in the optimized medium at 37 °C.

#### 3.6.2. Phage Propagation and Stock Production

To sufficiently propagate the phage, the purified phage solution was infected to the *S. aureus* ATCC 29213 culture grown for 1.5 h and further incubated aerobically for 4 h at 37 °C. The culture was then centrifuged at 15,000× *g* for 5 min, and the lysate was filtered through a Whatman^TM^ PVDF membrane filter. The medium volume was increased from 3 to 300 mL by repeating this procedure three times. The phage lysate was centrifuged at 30,000× *g* for 20 min to prepare the phage stock. Subsequently, the phage particles from which the supernatant was removed were dissolved in PBS. The highly concentrated phage stock was stored at 4 °C.

### 3.7. Characterization of the Phage PBSA08

#### 3.7.1. Transmission Electron Microscopy (TEM) Analysis

A formvar carbon-coated copper grid was used to hold the purified phage (10^9^ PFU/mL), which was then negatively stained with 2% (*v*/*v*) uranyl acetate. An energy-filtering Libra 120 electron microscope (Carl Zeiss, Oberkochen, Germany) at 120 kV of accelerating voltage was used to examine the morphological features of the phage. It was confirmed that the phage size was the average of five independent measurements.

#### 3.7.2. Bacteriolytic Activity

Exponentially growing *S. aureus* ATCC 29213 culture (4 × 10^8^ CFU/mL) in TSB (50 mL) was infected with phage solution (1 mL) at different multiplicity of infections (MOIs = 0.001, 0.01, 0.1, and 1). The culture was incubated aerobically for up to 24 h at 37 °C. The bacteriolytic activity was ascertained by measuring the absorbance (600 nm) of the culture every hour.

#### 3.7.3. Temperature and pH Stability

Phage stability was confirmed under a wide temperatures (−18 °C to 70 °C) and pH range (4–11). High concentrations (5 × 10^8^ PFU/mL) of phage were used in these experiments. To investigate thermal stability, PBS (990 μL) and phage solution (10 μL) were blended and incubated for 30 min at each temperature condition. To determine pH stability, pH buffer (990 μL) and phage solution (10 μL) were blended and incubated for 30 min at 25 °C.

#### 3.7.4. Host Range Determination

Phage host range was determined by spotting assay as described previously [22]. Briefly, 10-fold serial dilutions of phage solution (5 × 10^8^ PFU/mL) were spotted on the lawns of the target strain. The lysis sensitivity was validated by the presence of a plaque or inhibition zone on the lawns of the strains. The efficiency of plaquing (EOP) was determined by dividing the output phage titer on the target strain by the input phage titer on the host strain (*S. aureus* ATCC 29213).

### 3.8. Phage DNA Extraction and Genome Sequence Analysis

#### 3.8.1. Genomic DNA Extraction

Phage DNA was extracted according to modified phenol–chloroform protocols [19]. Briefly, DNase I and RNase I were used to treat the phage solution in order to eliminate any remaining bacterial DNA/RNA contamination. After being processed with lysis buffer [0.5 M EDTA, 0.5% SDS, and proteinase K], the purified phage was incubated for 15 min at 65 °C. After adding the same amount of phenol to the mixture, it was centrifuged at 5000 rpm for 5 min at RT. The supernatant layer was treated with phenol-chloroform-isoamyl alcohol (25:24:1) and chloroform using the same above-described method. After that, ethanol and sodium acetate were added to the supernatant for DNA precipitation. Finally, the phage DNA was condensed in TE buffer and stored at −18 °C.

#### 3.8.2. Genome Sequencing and Analysis

The open reading frames (ORFs) were analyzed using RAST (https://rast.nmpdr.org/, accessed on 15 July 2025) and FgenesV (Softberry Inc., Mount Kisco, NY, USA, http://www.softberry.com/berry.phtml, accessed on 15 July 2025). The ORFs were annotated using the BLAST of NCBI (https://blast.ncbi.nlm.nih.gov/Blast.cgi, accessed on 15 July 2025) and InterProScan (https://www.ebi.ac.uk/interpro/search/sequence/, accessed on 15 July 2025), followed by processing with an Artemis program (https://www.sanger.ac.uk/tool/artemis/, accessed on 15 July 2025). A genome map was constructed with Genescene (DNAstar, Madison, WI, USA). Phylogeny was analyzed based on the amino acid sequence of the major tail protein using MEGA 11. Phage PBSA08’s genome sequence is listed in GenBank with accession number OP856857.

### 3.9. Antibacterial Efficacy of the Phage-Coated Film

Antibacterial effects of the phage-coated film were spectroscopically evaluated in vitro. Film samples were sliced into circles with a diameter of 25 mm (10^6^ PFU/film) and placed into a 12-well microplate. Each well was added with 2 mL of TSB and inoculated with 20 μL of *S. aureus* ATCC 29213 (10^6^ CFU). Subsequently, the microplate was kept at 37 °C under aerobic conditions (120 rpm). Wells without the film were used as the control. Bacterial growth inhibition activity was observed by measuring the culture absorbance at 600 nm every hour for 24 h.

### 3.10. Statistical Analysis

The mean ± standard deviation from a minimum of three experiments was used to express the results. SPSS version 26 (SPSS Inc., Chicago, IL, USA) was used to perform a one-way analysis of variance on all the data. Duncan’s multiple-range test was used to assess significant differences between mean values, and data with a *p* value of <0.05 were deemed statistically significant.

## 4. Conclusions

A PLA/PCL composite film with enhanced miscibility was developed by adding the compatibilizer TBC that induces internal bonding between PLA and PCL and improves the moisture barrier by reducing micro-voids and phase separation. The combination of SA and glycerol exhibited optimal dynamic behavior as a coating solution and excellent adhesion to the film surface, indicating that the SA combination is a suitable phage delivery system and can be employed as a useful coating agent for plastic packaging. Antibacterial properties were imparted to the PLA/PCL/TBC film by coating it with SA containing *S. aureus* phage, and the developed film exhibited substantial bacterial growth inhibition. Overall, these findings demonstrate that films coated with antibacterial agents can be used as active packaging. To our knowledge, this study is the first to load phages into SA and coat them onto PLA/PCL/TBC films, and it is anticipated that this will provide a continuous and substantial antimicrobial effect through the regulated release of biological agents under actual packaging conditions. Although bar-coating was used to simulate pilot-scale coating, future work should consider scalable techniques such as gravure or slot-die coating for industrial production. These methods offer higher throughput and more precise coating control. In addition, as both SA and phages are biodegradable and non-toxic, the environmental effect of these materials may be very low. However, regulatory approval processes must ensure phage safety and batch consistency. Finally, the phage coating should be conducted under realistic packaging and disposal conditions to support regulatory acceptance.

## Figures and Tables

**Table 1:** Bacterial strains used in this study and host range result of phage PBSA08.

Bacterial Strain	Lysis Sensitivity ^a^	Source ^b^
*Staphylococcus aureus* strains		
ATCC 29213	+ + +	ATCC
ATCC 6538	+ + +	ATCC
ATCC 12600	+ + +	ATCC
ATCC 13301	+ +	ATCC
ATCC 23235	+ + +	ATCC
ATCC 25923	+ +	ATCC
ATCC 27664	+ + +	ATCC
ATCC 33586	+ + +	ATCC
ATCC 33593	+ +	ATCC
CCARM 3089	+ + +	CCARM
CCARM 3090	+	CCARM
CCARM 3793	+	CCARM
KCCM 12103	+	KCCM
KCTC 1916	+ + +	KCTC
Gram-negative bacteria		
*Escherichia coli* O157:H7 ATCC 43890	-	ATCC
*Klebsiella pneumoniae* KCTC 2242	-	KCTC
*Vibrio parahaemolyticus* KCTC 2471	-	KCTC
*Vibrio cholerae* NCCP 13589	-	NCCP
*Shigella flexneri* KCTC 2517	-	KCTC
*Pseudomonas aeruginosa* ATCC 27853	-	ATCC
*Yersinia enterocolitica* ATCC 55075	-	ATCC
*Salmonella enterica* Enteritidis ATCC 13076	-	ATCC
*Salmonella enterica* Typhimurium KCTC 1425	-	KCTC
*Pectobacterium carotovorum* KACC 21701	-	KACC
*Cronobacter sakazakii* ATCC 29544	-	ATCC
Gram-positive bacteria		
*Listeria monocytogenes* ATCC 15313	-	ATCC
*Bacillus cereus* ATCC 14579	-	ATCC
*Enterococcus faecalis* ATCC 19433	-	ATCC

^a^ + + +, EOP > 1; + +, EOP of 0.5–1; +, EOP < 0.5; -, no susceptibility to phage PBSA08. ^b^ ATCC, American Type Culture Collection; CCARM, Canadian Centre for Agri-Food Research in Health and Medicine; KCTC, Korean Collection for Type Cultures; NCCP, National Culture Collection for Pathogens; KACC, Korean Agricultural Culture Collection.

## Data Availability

All data are provided within this article.

## Supplementary Materials

The following supporting information can be downloaded at: https://www.mdpi.com/article/10.3390/ijms26167793/s1.
