# Supplementary material for: Development of an Antibacterial Poly(Lactic Acid)/Poly(ε-Caprolactone)/Tributyl Citrate Film Loaded with Staphylococcus aureus Bacteriophages Using a Sodium Alginate Coating
Source: Int J Mol Sci. 2025 Aug 12;26(16):7793. doi: 10.3390/ijms26167793 (PMC12386292; doi:10.3390/ijms26167793)
Supplement: Supplementary file 1 [file ijms-26-07793-s001.zip › ijms-3777089-supplementary.pdf]

Supplementary Materials

Table S1. Tensile strength and elongation at break of PLA/PCL blends

Film composition	Thickness (mm)	Tensile strength (MPa)	Elongation at break (%)
100/0	0.062 ± 0.001 ^{f}	30.89 ± 0.24 ^{a}	13.76 ± 0.54 ^{e}
90/10	0.065 ± 0.003 ^{e}	19.86 ± 1.74 ^{b}	20.94 ± 1.45 ^{c}
80/20	0.076 ± 0.002 ^{d}	16.68 ± 0.39 ^{d}	22.18 ± 2.68 ^{c}
70/30	0.079 ± 0.002 ^{c}	18.05 ± 0.06 ^{c}	33.08 ± 2.45 ^{b}
60/40	0.083 ± 0.006 ^{b}	7.05 ± 0.68 ^{f}	14.32 ± 2.30 ^{d}
0/100	0.085 ± 0.002 ^{a}	10.84 ± 0.79 ^{e}	116.96 ± 4.12 ^{a}

$^{a–f}$ Different capital letters in the same column indicate significant differences ($P < 0.05$).

Table S2. Tensile strength and elongation at break of PLA/PCL films with and without TBC

TBC concentration (w/v %, of PCL weight)	Thickness (mm)	Tensile strength (MPa)	Elongation at break (%)
0	0.065 ± 0.003 ^{e}	19.86 ± 1.74 ^{d}	20.94 ± 1.45 ^{b}
5	0.067 ± 0.002 ^{d}	21.93 ± 2.33 ^{cd}	21.02 ± 1.54 ^{b}
10	0.068 ± 0.003 ^{c}	24.31 ± 2.01 ^{c}	21.08 ± 1.61 ^{b}
15	0.071 ± 0.006 ^{b}	27.18 ± 1.90 ^{b}	21.20 ± 1.03 ^{b}
20	0.078 ± 0.003 ^{a}	30.09 ± 0.80 ^{a}	23.34 ± 1.43 ^{a}

$^{a–e}$ Different capital letters in the same column indicate significant differences ($P < 0.05$).

Table S3. Surface color and transmittance values of PLA/PCL films with and without TBC

Films	L^*	a^*	b^*	ΔE	T_{280} (%)	T_{660} (%)
PLA	92.17 ± 0.20 ^{c}	−0.23 ± 0.03 ^{b}	4.57 ± 0.21 ^{a}	0.39 ± 0.23 ^{a}	61.57 ± 0.85 ^{a}	93.69 ± 0.31 ^{a}
PCL	92.97 ± 0.13 ^{a}	−0.21 ± 0.02 ^{b}	4.49 ± 0.21 ^{a}	0.54 ± 0.18 ^{bc}	0.93 ± 0.06 ^{c}	4.92 ± 0.15 ^{d}
PLA_{90}/PCL_{10}	92.55 ± 0.19 ^{b}	−0.28 ± 0.04 ^{a}	4.51 ± 0.07 ^{a}	0.27 ± 0.06 ^{b}	44.59 ± 0.21 ^{b}	84.91 ± 0.14 ^{b}
$PLA_{90}/PCL_{10}/TBC_5$	92.73 ± 0.14 ^{b}	−0.27 ± 0.02 ^{a}	4.51 ± 0.12 ^{a}	0.30 ± 0.18 ^{c}	45.04 ± 0.41 ^{b}	84.31 ± 0.21 ^{c}

$^{a–d}$ Different capital letters in the same column indicate significant differences ($P < 0.05$).

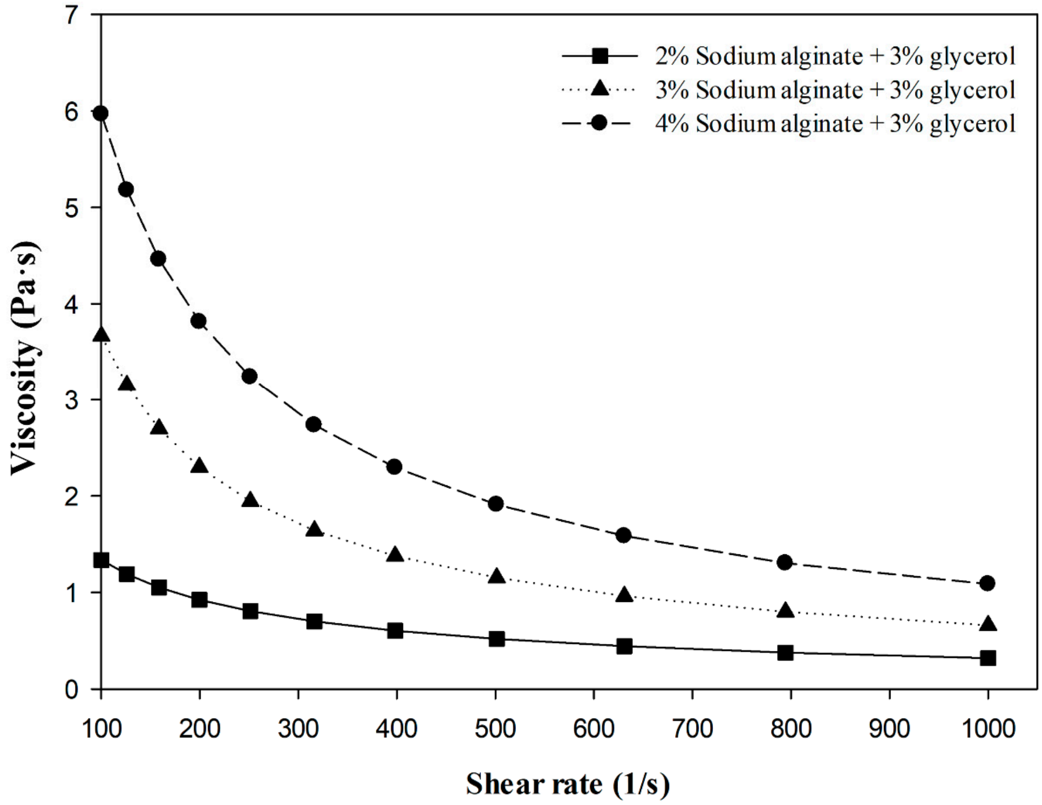

Figure S1. Viscosity curve of sodium alginate (SA) coating solutions at different concentrations. ■, 2% SA + 3% glycerol; ▲, 3% SA + 3% glycerol; ●, 4% SA + 3% glycerol. Error bars indicate the standard deviation.
